# An Optical Interferometric Triaxial Displacement Sensor for Structural Health Monitoring: Characterization of Sliding and Debonding for a Delamination Process

**DOI:** 10.3390/s17112696

**Published:** 2017-11-22

**Authors:** Chen Zhu, Yizheng Chen, Yiyang Zhuang, Yang Du, Rex E. Gerald, Yan Tang, Jie Huang

**Affiliations:** 1 Department of Electrical and Computer Engineering, Missouri University of Science and Technology, Rolla, MO 65409, USA; cznwq@mst.edu (C.Z.); ycb28@mst.edu (Y.C.); yz8r4@mst.edu (Y.Z.); duya@mst.edu (Y.D.); yty93@mst.edu (Y.T.); 2 American Inventor Institute, Willow Spring, IL 60480, USA; rexgeraldii@gmail.com

**Keywords:** three-dimensional displacement, extrinsic Fabry–Perot interferometer, interfacial sliding, debonding, structural health monitoring

## Abstract

This paper presents an extrinsic Fabry–Perot interferometer-based optical fiber sensor (EFPI) for measuring three-dimensional (3D) displacements, including interfacial sliding and debonding during delamination. The idea employs three spatially arranged EFPIs as the sensing elements. In our sensor, the three EFPIs are formed by three endfaces of three optical fibers and their corresponding inclined mirrors. Two coincident roof-like metallic structures are used to support the three fibers and the three mirrors, respectively. Our sensor was calibrated and then used to monitor interfacial sliding and debonding between a long square brick of mortar and its support structure (i.e., a steel base plate) during the drying/curing process. This robust and easy-to-manufacture triaxial EFPI-based 3D displacement sensor has great potential in structural health monitoring, the construction industry, oil well monitoring, and geotechnology.

## 1. Introduction

Over the years, steel-reinforced concrete (RC) has been extensively used in civil infrastructures [1]. Throughout the life cycle of a reinforced concrete structure, many issues must be properly addressed to assess the safety and serviceability of the structure [2].

The interfacial bonding between the concrete and steel is always a concern. The debonding due to shrinkage of concrete or corrosion of steel can decrease the tensile strength and ductility of the RC, leading to a catastrophic failure of the structures [3]. Additionally, the relative sliding (horizontal and vertical directions) between the concrete and steel, yielded by the insufficient shear bond strength and irregular deformation, can directly destroy the integrity of RC, causing deterioration of the interior stress state [4]. Therefore, it is very important to detect both sliding and debonding in RC in time to ensure its safety and determine its fitness for service. A number of sliding and debonding sensors have been developed with various sensing mechanisms, primarily including mechanical, electrical, and acoustic [1,2,3,5,6,7]. Acoustic emission (AE) is one of the most powerful tools to assess the integrity of the RC structure, which correlates the AE parameters with the localized changes inside the structure [5]. Another widely used technique is the piezoelectric-based approach, which is based on impedance analysis or comparisons of vibration-characteristics [8]: the operational principle of the first method is based on the electromechanical coupling properties of piezoelectric materials; the latter method employs piezoelectric transducers to actively excite and sense the vibrational characteristics of the structure, and then compares the vibrational signatures with those of a healthy-state structure. However, these kinds of sensors have demanding requirements for a stable and interference-free working environment. For instance, large temperature fluctuations, strong electromagnetic interference, and other harsh environmental factors can have significant detrimental influences on the performance of these sensors.

In recent years, optical fiber sensors have attracted considerable attention and have been widely used for structural health monitoring (SHM) [9,10]. In comparison to traditional sensing technologies, optical fiber sensors offer great advantages, such as immunity to electromagnetic field, high sensitivity, small size, ease of fabrication, and robustness to harsh environments [11,12,13,14]. Given several choices of sensing mechanisms, such as wavelength modulation, phase modulation, and intensity modulation, optical fiber displacement sensors thrive on structural diversity and sensing properties [15,16,17,18,19,20,21,22,23,24]. The majority of the reported optical fiber displacement sensors work in a one-dimensional fashion, meaning it is impossible to measure interfacial sliding and debonding at the same time [15,16,17,18,19,20]. Several two- and three-dimensional (2D, 3D) optical fiber displacement sensors have been demonstrated [21,22,23]. Zhu et al. developed fiber Bragg grating (FBG) sensing bars for monitoring internal displacements of a 2D model dam [21]. Rapp et al. reported a displacement field estimation for a 2D structure using fiber Bragg grating sensors [23]. Despite the great efforts that have been made in the development of multidimensional optical fiber displacement sensors, accurate measurements of interfacial sliding and debonding still remain a great challenge. This is because the aforementioned multidimensional sensors operate based on the modal transformation method to reconstruct displacements from measured strains [23]. What these sensors can measure is the deformation of one object (e.g., the concrete), rather than the relative displacements between two objects (e.g., between concrete and steel), both of which can deform. Therefore, the sliding and debonding cannot be accurately determined at the same time.

An extensively studied interferometric sensor, the extrinsic Fabry–Perot interferometer (EFPI), has the merit of measuring displacements. An EFPI is generally formed by the endface of an optical fiber and an external reflecting surface [24]. The distance between the two reflecting surfaces can directly affect the reflection spectrum of an EFPI [24]. With proper structure design and packaging, EFPI sensors could be considered excellent candidates for accurately measuring displacements [14,18,24,25].

In this paper, we demonstrate a stable triaxial EFPI-based optical fiber sensor for 3D relative displacement measurements, including interfacial sliding and debonding, useful for describing the delamination process. In our sensor design, two roof-like metallic structures are used to support the optical fibers and mirrors, ensuring that the endfaces and their corresponding mirrors are parallel. Three EFPIs are formed by the endfaces of optical fibers and their corresponding mirrors. When there are 3D relative displacements between the two roof-like metallic structures, the cavity lengths of the EFPIs will change correspondingly. Our sensor was firstly calibrated to determine the cavity length-displacement coefficient matrix for the sensor. Then, the utility of the triaxial displacement sensor was demonstrated by monitoring interfacial sliding and debonding between a sample of mortar and a steel base plate during the drying/curing process of the mortar.

## 2. Sensor Structure and Principle

**Figure 1:**
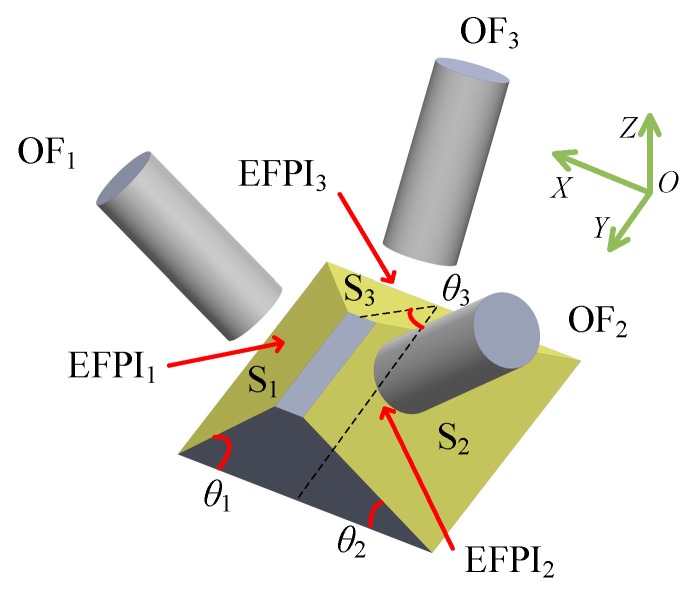
A schematic diagram of the triaxial displacement sensor. The sensor consists of an optical fiber component and a mirror component. OF represents optical fiber. Three EFPIs are formed by the endfaces of the three optical fibers and their corresponding mirrors. The inclined surfaces S_1_, S_2_, and S_3_ have angles of inclination *θ*_1_, *θ*_2_, and *θ*_3_ (0° < *θ*_1_, *θ*_2_, *θ*_3_ < 90°) with respect to the *XOY* plane, respectively. A 3D coordinate system, *XYZ*, is defined in the figure.

A schematic drawing of the sensor is illustrated in Figure 1, including the optical fiber and mirror components, each of which is fixed to a separate roof-like supporting body. The fiber component consists of three optical fibers and their supporting body, and the mirror component consists of three mirrors and their supporting body. The two roof-like supporting bodies can move independently in the 3D domain. The inclined mirror surfaces S_1_, S_2_, and S_3_ have angles of inclination *θ*_1_, *θ*_2_, and *θ*_3_ (0° < *θ*_1_, *θ*_2_, *θ*_3_ < 90°) with respect to the *XOY* plane, respectively. The flat metal surfaces of the mirror component are each coated with a gold layer with a thickness of approximately 600 nm using the Thermal Evaporation Deposition method, each mirror achieving a reflectivity of 99%. The size of each mirror is approximately 10 mm^2^. The endfaces of the optical fibers are parallel to the corresponding mirrors. As a result, three EFPIs are formed by the endfaces of three optical fibers and their corresponding mirrors. According to the established geometric relationships, the relative displacements in a 3D domain between the optical fiber component and the mirror component can be effectively compressed and transferred to variations of cavity lengths of the formed EFPIs. For instance, when there is a relative sliding along the *X* direction, the cavity lengths of EFPI_1_ and EFPI_2_ will change. The displacements Δ*x*, Δ*y*, and Δ*z* of the optical fiber component with respect to the mirror component in a 3D domain can be calculated by

(1) $$\left\{\begin{array}{c} \Delta x\\ \Delta y\\ \Delta z \end{array}\right\}={\left[\begin{array}{ccc} \sin\theta_{1} & 0 & \cos\theta_{1}\\ -\sin\theta_{2} & 0 & \cos\theta_{2}\\ 0 & -\sin\theta_{3} & \cos\theta_{3} \end{array}\right]}^{-1}\left\{\begin{array}{l} \Delta L_{1}\\ \Delta L_{2}\\ \Delta L_{3} \end{array}\right\}$$

where Δ*L*_1_, Δ*L*_2_, and Δ*L*_3_ are the changes in the cavity lengths of the three EFPIs, respectively. It should be noted that the cavity length-displacement coefficient matrix should be adjusted if the 3D coordinate system is changed. Since all three EFPIs function based on the same principle, we will only discuss the sensing and demodulation mechanism for one of them (e.g., EFPI_1_).

As stated, EFPI_1_ with a cavity length of *L*_1_ is formed by the endface of OF_1_ and the mirror mounted on S_1_. An interference pattern is generated by the two reflected beams. The intensity for the superposition of the two waves, the interference signal *I*_1_, is given by

(2) $$I_{1}=I_{11}+I_{12}+2\sqrt{I_{11}I_{12}}\cos(\frac{4\pi L_{1}}{\lambda }+\varphi ).$$

In Equation (2), *I*_11_ and *I*_12_ denote the reflected light intensities from the endface of OF_1_ and the corresponding mirror S_1_, respectively; *ϕ* is the initial phase difference of the interferometer. The difference in wavelength between two successive minima in the spectrum, defined as the free spectral range (*FSR*) [26], can then be expressed as

(3) $$FSR_{1}=\lambda_{1b}-\lambda_{1a}=\frac{\lambda_{1a}\lambda_{1b}}{2L_{1}}$$

where *λ*_1*a*_ and *λ*_1*b*_ are the wavelengths corresponding to the two peak points in the interference spectrum of EFPI_1_; *FSR*_1_ is the *FSR* of the interference spectrum of EFPI_1_; *L*_1_ is the cavity length of EFPI_1_. According to Equation (3), the cavity length *L*_1_ can be calculated. With reference to the demodulation principle described above, variations in the cavity lengths of the three EFPIs can be determined. Therefore, the displacements in the 3D domain can be calculated by Equation (1). It should be noted that a displacement transfer mechanism is employed in the sensor design [14]. The user-configurable, triangle-geometry-based displacement transfer mechanism provides a means for adjusting the inclination of the mirrors to specify the dynamic range for each of the three displacement sensors and is described in detail in [14].

## 3. Experimental Results and Discussion

### 3.1. Sensor Characterization

**Figure 2:**
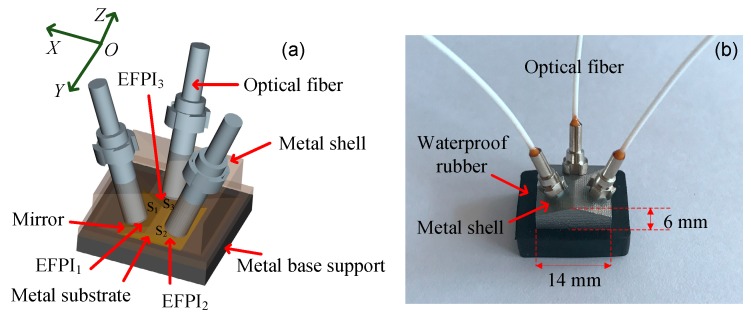
(**a**) Three-dimensional rendering of the triaxial sensor model for 3D relative displacement sensing. The mirrors and endfaces of the corresponding optical fibers are parallel, such that three EFPIs are formed. Both the optical fibers and mirrors are supported on separate roof-like structures. (**b**) A photograph of the sensor. The sensor is packaged and protected by a metal shell and a waterproof rubber casing. The size of the metal shell is 14 mm × 14 mm × 6 mm.

The structure of the triaxial displacement sensor is shown in Figure 2a. Three optical fibers, mounted in ceramic ferrules, are inserted into a roof-like metal shell component through the holes on the three sloping sides, respectively. Three gold surface mirrors are mounted on a similar roof-like metal shell component, which is underneath the optical fiber metal shell component. As a result, three EFPIs with air-gaps are formed by the endfaces of the optical fibers and corresponding mirrors. The sensor structure is packaged in a metal shell, which protects the space containing the EFPIs dust-free. The metal base support is used to fix the mirror component. Figure 2b presents a photograph of our triaxial displacement sensor. Our triaxial sensor provides high measurement resolution, up to a level of tens of nanometers. The fiber component and the mirror component can be embedded in or attached to different adjacent objects (e.g., mortar and steel plate) for measuring 3D relative displacements between the different structural elements.

**Figure 3:**
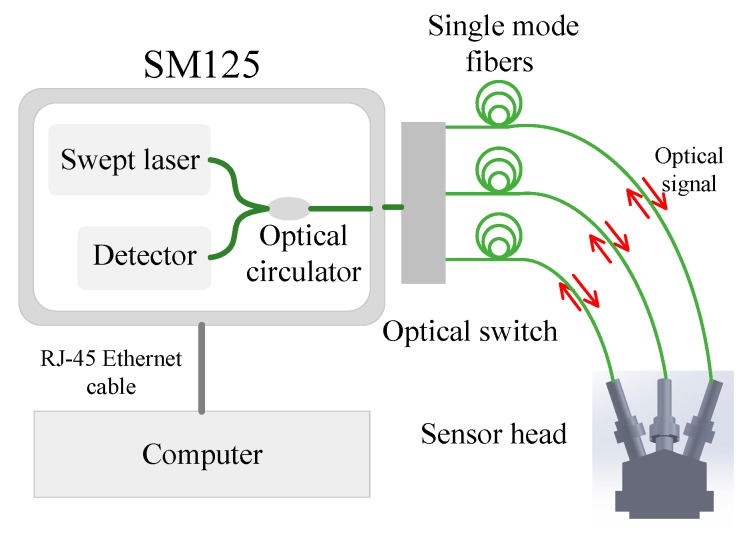
A schematic diagram of the experimental setup. Three input–output channels are connected to the three single mode fibers, which terminate and form three EFPIs in the sensor head. An integrated optical switch is used for rapid switching between EFPIs.

The experimental setup used for the calibration of the triaxial displacement sensor is illustrated in Figure 3. A wavelength interrogator/demodulator (SM125, Micron Optics, Inc., Atlanta, GA, USA) is used as the light source, detector, and demodulator. The interrogator has four optical channels and an internal optical switch that allows computer-controlled switching between the four channels. The incident light beam from the swept laser is directed to each of the EFPI sensors in a continuous round-robin sequence by the optical switch. The reflected interference signal described by Equation (2) consists of a reflected beam from the end face of the optical fiber and a reflected beam from the corresponding inclined gold mirror. The interference signal is routed through the optical circulator to the detector. The interference spectrum is obtained by sweeping the wavelength of the laser from 1510 to 1590 nm in equal intervals (i.e., 0.005 nm) and by recording the corresponding intensities of the reflected signals. The update rate of the interrogation unit can be up to 1 Hz. A personal computer is connected to the interrogator using an RJ-45 Ethernet (Micron Optics, Inc., Atlanta, GA, USA) cable. Bi-directional communication between the computer and the SM125 follows the TCP/IP protocol, and is used to set acquisition parameters, acquire the data, and store the data. The personal computer is also used to process the interference spectra. The cavity length of each of the EFPIs can then be determined using a LabVIEW-based data processing program employing the demodulation mechanism described in [25]. It should be noted that an intensity-based demodulation system can also be used for the proposed sensor. If a single-wavelength light source is used, the output intensity of the reflected wave will be a sinusoidal function of the cavity length, which is the superposition of the two light beams, one reflected by the optical fiber endface and the other by the corresponding mirror. However, the measurement range of the single-wavelength light source based intensity system will be limited—less than a wavelength (i.e., 1.5 μm)—since the linear response region of the sensor will be the rising/falling curve of the sinusoidal function.

**Figure 4:**
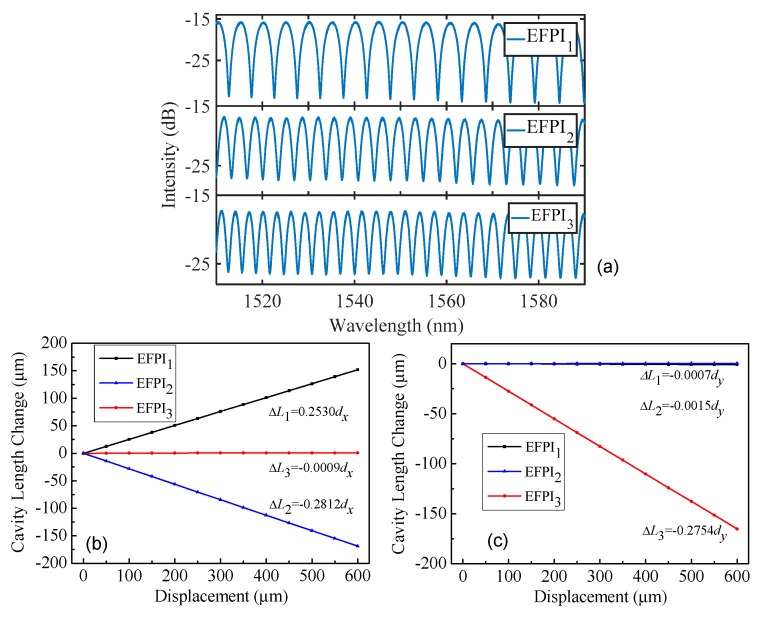
Calibration results of the triaxial displacement sensor. (**a**) Interference spectra recorded from the triaxial displacement sensor for the initial cavity length settings. (**b**) The changes in cavity lengths of the three EFPIs as a function of applied displacements along the *X* direction (*d_x_*). (**c**) The changes in cavity lengths of the three EFPIs as a function of applied displacements along the *Y* direction (*d_y_*). In the experiment, the mirror component was fixed on an optical table, and the optical fiber component was positioned by an optical stage. The coordinate system used in the experiment is the same as the *XYZ* system shown in Figure 2a.

Figure 4a shows the interference spectra of the triaxial sensor at the initial cavity length settings. The initial cavity lengths of the three EFPIs were 233.725 μm, 338.913 μm, and 381.935 μm, respectively. We report results for displacement calculations that contain six significant figures because an EFPI can typically resolve a 1 nm change in cavity length. In our calibration experiment, the mirror component was fixed on an optical table, and the optical fiber component was positioned using an optical translation stage. The calibration experiment was conducted at room temperature. The coordinate system used in the experiment was the same as that shown in Figure 2a. Displacements along the *X* and *Y* directions (*d_x_*, *d_y_*) were applied to the optical fiber component of the sensor. The magnitudes of the applied displacements were accurately determined using a Mitutoyo micrometer (resolution, 0.1 μm; dynamic range, 25 mm) [14]. The relationships between the displacements and the corresponding changes in cavity lengths of the EFPIs for a sequence of changes along the two different directions are shown in Figure 4b,c. The results matched well with theoretical expectations. When the optical fiber component of the sensor was subjected to a displacement along the *X* direction, the cavity lengths of EFPI_1_ and EFPI_2_ changed in opposite directions while that of EFPI_3_ did not change. On the contrary, when the optical fiber component moved along the *Y* direction, the cavity length of EFPI_3_ varied with the applied displacement, while the cavity lengths of EFPI_1_ and EFPI_2_ remained constant. The small differences between the theoretical and experimental results are ascribed to machining errors for the flatness of the sloping sides of the mirror component of the sensor. According to the calibration results shown in Figure 4b,c, the inclinations of the three mirrors were calculated to be 14.656°, 16.332°, and 15.987°, respectively. The coefficient matrix in Equation (1) that describes the relationships between changes in the cavity lengths and the corresponding 3D displacements for the sensor is calculated to be ${\left[\begin{array}{ccc} 0.2530 & 0 & 0.9675\\ -0.2812 & 0 & 0.9596\\ 0 & -0.2754 & 0.9613 \end{array}\right]}^{-1}$. The dynamic ranges in the *X*, *Y*, and *Z* directions of the sensor are designed to be ±1.5 mm, ±1.5 mm, and 1.0 mm, respectively. As mentioned above, the dynamic ranges can be flexibly modified by designing the inclinations of the three mirrors and the package size of the sensor.

**Figure 5:**
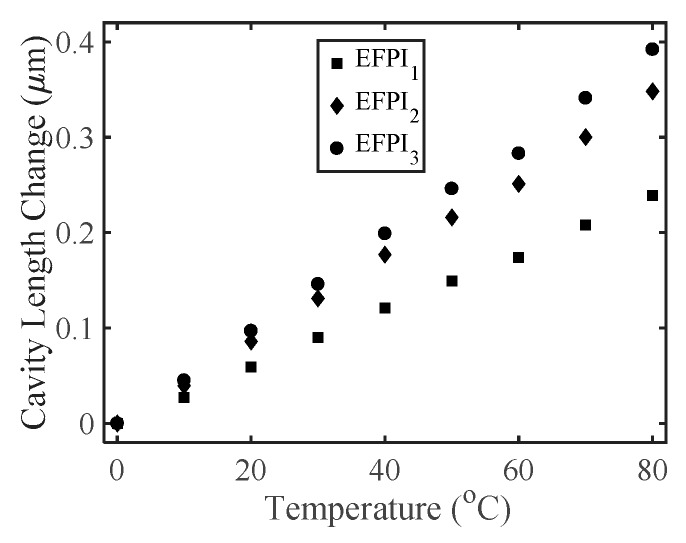
Measured cavity length change of EFPI_1_, EFPI_2_, and EFPI_3_ with respect to temperature.

The temperature response of the triaxial displacement sensor was also investigated by placing the sensor in a temperature-controlled box (±1 °C, KEEN BING) [14]. The measured temperature response of the sensor is shown in Figure 5. Linear curve fits were applied to the three measured data sets, and the results showed that the temperature sensitivities of the EFPI_1_, EFPI_2_, and EFPI_3_ were 0.00298 μm/°C, 0.004314 μm/°C, and 0.00488 μm/°C, respectively. The three slopes for a 1 °C change are fairly small compared to the cavity lengths of the three EFPIs. However, in field applications where the ambient temperature could change by tens of degrees, the temperature influence on the sensor for displacement measurements should be compensated. For instance, an FBG could be inserted in the lead-in single-mode fiber for real-time compensation of changes in the cavity lengths due to temperature fluctuations. The corrections to the cavity lengths of the three EFPIs would be based on the measured temperature dependence coefficients.

### 3.2. Sensor Testing

**Figure 6:**
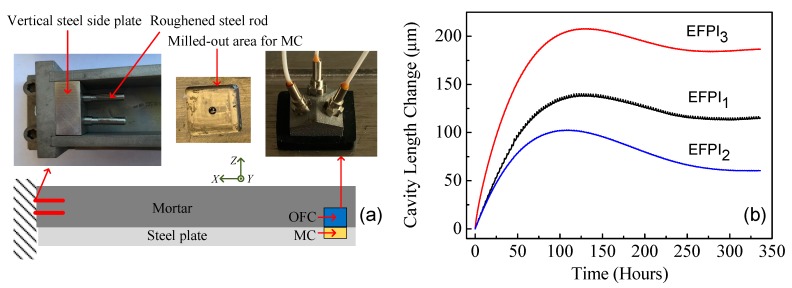
Continuous displacement measurements. (**a**) The schematic diagram of the experiment setup for monitoring delamination and sliding. Note that the mirror component (MC) is embedded in a milled-out square area that is 3 mm deep so that the top surface of the metal base support of the MC matches the top surface of the steel plate. (**b**) Cavity length changes as a function of time from three EFPIs during continuous displacement measurements for a brick of mortar during the drying/curing process. OFC and MC represent the optical fiber component and the mirror component, respectively. Mortar components and weight ratios: Sakrete Portland Type-1 cement, 1.0; tap water, 0.5; sand, 2.8. Brick of mortar size: 254.0 mm × 25.4 mm × 25.4 mm.

To demonstrate the practicability of the sensor, an experiment designed to monitor the interfacial sliding and debonding between a long square brick of mortar and a steel base plate during the drying/curing process of the mortar was conducted. The schematic diagram of the experiment is shown in Figure 6a. For the experiment, the mortar was prepared using cement (Sakrete Portland Type-I), tap water, and sand with a weight ratio of 1.0:0.5:2.81. The size of the long square brick of mortar was 25.40 × 2.54 × 2.54 cm. Prior to conducting the test experiment, a square section, 2 cm by 2 cm and 3 mm deep, was machined on the top of the steel base plate, which was used to mount and fix (using a machine screw) the mirror component of the sensor. It is important that the top surface plane of the metal base support of the mirror component matches the top surface plane of the steel plate for accurate measurement of interfacial sliding and debonding. The optical fiber component was mounted right above the mirror component, and a waterproof rubber cover was used to protect the sensor structure, as shown in Figure 6a. The next step was to pour the cement. As a result, the mirror component (fixed to the steel plate with a machine screw) shared a common surface with the steel plate, while the optical fiber component was integrated within and bonded to the concrete. Thus, the relative displacements between the concrete and the steel base plate could be measured by our sensor. We added a thin film of oil between the brick of mortar and the steel base plate to ensure weak interfacial bonding. The experiment began after the mortar was cured for one day. During the initial curing period, one end of the brick of mortar was rigidly bonded to the vertical steel side plate using two roughened steel rods fixed to the vertical steel side plate and protruding into the brick of mortar, as shown in Figure 6a.

Figure 6b presents the experimental results for monitoring the interfacial sliding and debonding between the long brick of mortar and the steel base plate. During the mortar shrinkage process, the relative 3D displacements were metered by our sensor and interpreted as interfacial sliding along the *X* direction, buckling along the *Y* direction, and debonding along the *Z* direction between the brick of mortar and the steel base plate. The monitoring experiment lasted for 14 days. Throughout the period of the experiment, the mortar/steel structure was contained in a temperature-controlled box. According to the experimental results presented in Figure 6b, our sensor operated continuously for a long period of time, which is a key factor in practical applications.

**Figure 7:**
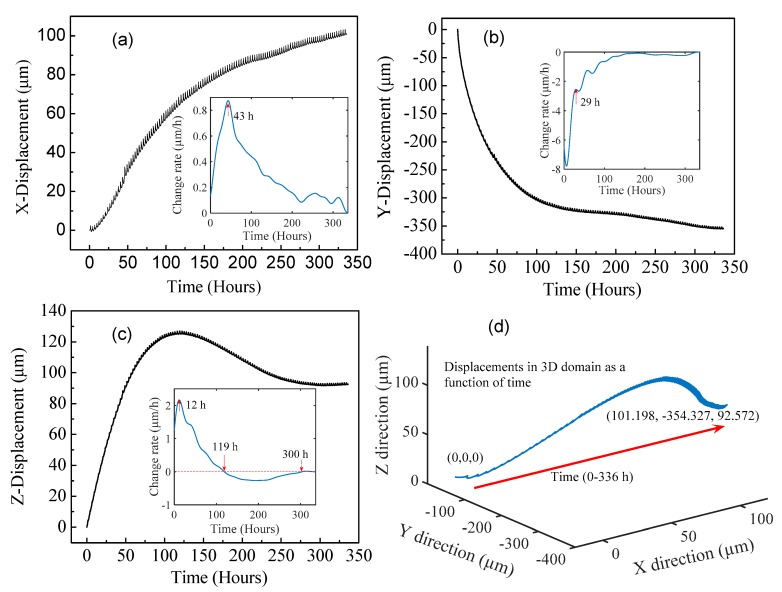
Real-time monitoring of 3D displacements between the long square brick of mortar and the steel base plate during the mortar curing/drying process. (**a**–**c**) Displacements versus time data for the *X*, *Y*, *Z* coordinates, respectively. The insets are plots of the time derivatives of the displacements as a function of measurement time for the *X*, *Y*, and *Z* directions. (**d**) Measured displacements plotted in the 3D domain parameterized by a time variable. The coordinate system used in the experiment was the same as the *XYZ* coordinate system shown in Figure 5a. The displacements along the *X*, *Y*, and *Z* directions revealed the interfacial sliding along the *X* direction, the buckling along the *Y* direction, and the debonding along the *Z* direction between the mortar and the steel base plate, respectively.

Using the transformation coefficient matrix, the interfacial sliding and debonding described in the 3D domain can be calculated and plotted, as shown in Figure 7. The insets in Figure 7a–c are plots of the time derivatives of the displacements along the *X*, *Y*, and *Z* directions with respect to time, respectively. The time derivatives were calculated after a low-pass filter was applied to the displacement vs. time data for the *X*, *Y*, and *Z* directions. The displacement along the *X* direction, representing the shrinkage along the long axis of the brick of mortar, increased with time. The *X*-displacement reveals a sliding between the brick of mortar and the steel base plate. The magnitude of the displacement along the +*X* direction is due in part to the rigid interfacial bond between the vertical steel side plate and the brick of mortar. The time rate of change in the *X*-displacement peaks at approximately 43 h into the drying/curing process; we anticipate that this peak displacement rate is a function of the composition, geometry, configuration, etc. of the brick of mortar specimen. Along the negative *Y* direction, the magnitude of the displacement initially increased dramatically with time. The *Y*-displacement data reveals a buckling effect, amplified by the attachment of the brick of mortar to the vertical steel side plate. The time rate of change of the *Y*-displacement drops dramatically in the first 29 h of the drying/curing process and depicts the complex dynamics of compression buckling. The displacement along the positive *Z* direction firstly increased, then decreased, and finally flatted with time. The displacement along the positive *Z* direction represents debonding between the brick of mortar and the steel base plate. The time rate of change of the *Z*-displacement increases rapidly and peaks in the first 12 h of the drying/curing process. Interestingly, the time rate of change in the *Z*-displacement as a function of time reveals that the debonding between the brick of mortar and the steel base plate turned negative at 119 h, meaning that the gap size between the brick of mortar and the steel base plate started collapsing. It is conceivable that the non-monotonic displacements along the *Z* direction were the consequences of combined effects from both the shrinkage of the mortar and the constant force of gravity. Beyond 300 h, the gravitational force and the shrinkage of the mortar along the *Z* direction reached a balance (note that the *X*-displacement continues to increase beyond 300 h, indicating that the drying/curing process is not yet complete). As shown in Figure 7d, the position of the optical fiber component changed from the initial point (0, 0, 0) to the final position (101.198 μm, −354.327 μm, and 92.572 μm) at the conclusion of the experiment. The small ripples in the displacement curves were caused by the periodic temperature variations in the temperature-controlled box (±1 °C, KEEN BING), due to the regulation of refrigeration (around 2.9-h cycle), which matched well with the time interval between the two peaks of the ripples. Please note that the small ripples represent the temperature-induced fluctuations in the dimensions of the brick of mortar and the steel base plate. As can be seen, due to the long length of the concrete and base plate in the *X* direction (i.e., 25.4 cm), the amplitude of the small ripples in the *X*-displacement (i.e., Figure 7a) is larger than the *Y*- and *Z*-displacements. For example, a 1 °C change in temperature could induce an approximately 3 μm relative displacement in the *X* direction between the base plate and the concrete.

**Figure 8:**
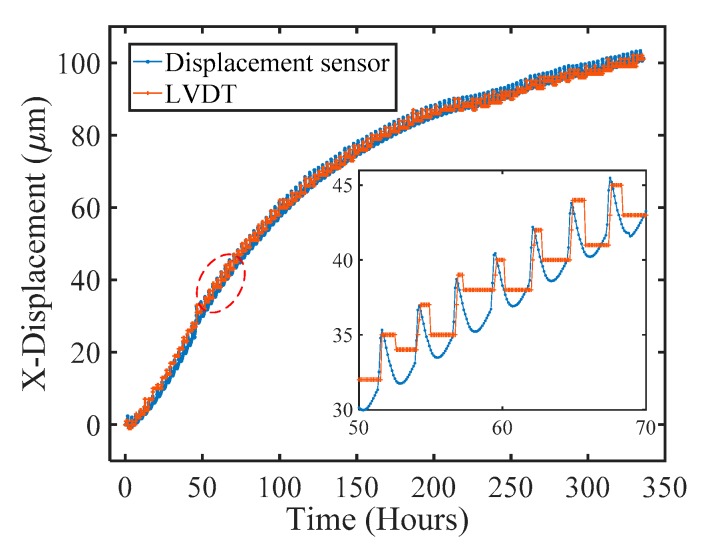
The displacement along the *X* direction measured by our prototype sensor and the LVDT. The inset shows the data collected during a period between 50 and 70 h.

During the experiment, the displacement along the *X* direction was also monitored using a commercial linear variable displacement transducer (LVDT) for comparison. Figure 8 shows a plot of the *X* displacement as a function of time measured by our prototype sensor and the LVDT. As can be seen, the results match well. However, it is obvious that our displacement sensor has a much better measurement resolution than the LVDT, and it can perfectly quantify the displacement evolution process with a continuous and reliable output, while the LVDT data only shows discrete data points displaced back and forth every micrometer. An analogous setup and process can be used to check and qualify the displacements in the *Y* and *Z* directions.

## 4. Conclusions

In conclusion, we invented, fabricated, and demonstrated a low-cost and robust prototype of an optical fiber sensor for measuring relative displacements in the 3D domain. The fundamental idea employs three spatially arranged EFPIs as a combined triaxial sensing device. In our sensor design, the three EFPIs are formed by the endfaces of three optical fibers and three corresponding parallel mirrors. The optical fibers collectively and the mirrors collectively are supported by independent roof-like metal structures. The optical fiber component is suspended above the mirror component. When there are 3D relative displacements between the two roof-like metallic structures, the cavity lengths of the EFPIs will change. A displacement transfer scheme that is user configurable and based on the geometry of a triangle was employed to adjust the dynamic range of each of the three EFPI sensors. Our triaxial sensor was calibrated and a cavity length-displacement coefficient matrix was calculated. Then, monitoring interfacial sliding and debonding between a long square brick of mortar and a steel base plate during the drying/curing process of the mortar was conducted to demonstrate the practicability of the triaxial displacement sensor. The experimental results show that our sensor is capable of accurately measuring the dynamics of the sliding and debonding process between a brick of mortar and the steel base plate, revealing interesting time points throughout the drying/curing process where the time rates of change for displacements peaked and plateaued. This resilient and simple-to-manufacture 3D displacement sensor has great potential for applications in SHM, the construction industry, oil well monitoring, geotechnology, and a wide range of other practical areas.
